# Analysis of Interfacial Adhesion Properties Between PBT Azide Propellant Matrix and Defective AP Fillers Using Molecular Dynamics Simulations

**DOI:** 10.3390/polym16243497

**Published:** 2024-12-15

**Authors:** Xianzhen Jia, Linjing Tang, Ruipeng Liu, Hongjun Liao, Liang Cao, Xianqiong Tang, Peng Cao

**Affiliations:** 1 National Key Laboratory of Energetic Materials, Xi’an Modern Chemistry Research Institute, Xi’an 710065, China; jiaxz1027@163.com (X.J.); tanglj22@163.com (L.T.); news_arrival@163.com (R.L.); 2 School of Mechanical Engineering and Mechanics, Xiangtan University, Xiangtan 411105, China; 202221572238@smail.xtu.edu.cn (H.L.); tangxq85@xtu.edu.cn (X.T.); 3 College of Architecture and Civil Engineering, Beijing University of Technology, Beijing 100124, China; liangcao988@126.com

**Keywords:** interfacial adsorption, defective packing, crosslinking rate, all-atom molecular dynamics simulation

## Abstract

Filler defects and matrix crosslinking degree are the main factors affecting the interfacial adhesion properties of propellants. Improving adhesion can significantly enhance debonding resistance. In this study, all-atom molecular dynamics (MD) simulations are employed to investigate the interfacial adsorption behavior and mechanisms between ammonium perchlorate (AP) fillers and a poly(3,3-bis-azidomethyl oxetane)-tetrahydrofuran (PBT) matrix. This study focuses on matrix crosslinking degree (70–90%), AP defects (width 20–40 Å), and temperature effects (200–1000 K) to analyze microscopic interfacial adsorption behavior, binding energy, and radial distribution function (RDF). The simulation results indicate that higher crosslinking of the PBT matrix enhances interfacial adsorption strength, but incomplete crosslinking reduces this strength. Defects on the AP surface affect interfacial adsorption by altering the contact area, and defects of 30 Å width can enhance adsorption. The analysis of temperature effects on binding energy and interface RDF reveals that binding energy and interface RDF fluctuate as the temperature increases. This study provides a microscopic perspective on the PBT matrix–AP interfacial adsorption mechanism and provides insights into the design of PBT azide propellant fuels.

## 1. Introduction

Azide polyether propellants are high-energy solid propellants widely used in spacecraft and missile applications. One of the most prominent azide propellant types is PBT-based composite propellants, which are synthesized from a random copolymer of PBT crosslinked with 2,6-toluene diisocyanate (TDI), and networked using trimethylolpropane (TMP) and triethylene glycol (TEG). The mechanical properties of these propellants are closely related to their crosslinking rate [1]. By optimizing component ratios, the performance of PBT-based azide propellants has been significantly improved [2].

AP, a white crystalline inorganic compound with a density of 1.95 g/cm^3^, exhibits significant oxidative characteristics. It can initiate combustion and explosive reactions when mixed with carbon-containing materials, organic substances, sulfur, phosphorus, or metal powders. Due to its high energy density and superior combustion properties, AP has been widely utilized in propellant applications and has become a focal point of research. PBT, as a high-performance propellant matrix, boasts advantages such as high heat generation, low glass transition temperature, high nitrogen content, high density, and low smoke emission. The crosslinked network structure of PBT plays a decisive role in its mechanical properties, and the crosslinking ratio and mechanism of PBT have emerged as key areas of research [1].

PBT-based composite propellants mainly consist of a PBT binder and AP solid filler, comprising over 80% of the propellant’s total mass. In AP/PBT propellants, the interfacial interactions between the solid filler and the binder are critical factors affecting the mechanical properties of the propellant [3,4,5,6]. Studying the mechanical properties of the PBT-AP interface is essential for understanding adhesion mechanisms and optimizing propellant performance. The term “interfacial interaction capability” refers to the ability of an adhesive to form strong bonds on the surface of fillers through various mechanisms such as hydrogen bonding and van der Waals (vdW) forces. Stronger interfacial interactions generally lead to better overall mechanical performance of the propellant.

Over the years, numerous experimental studies have been conducted on the PBT/AP interface [7,8,9,10,11,12]. These studies show that the key factors affecting interfacial adhesion include the crystallographic plane of filler, the molecular structure of the matrix, the interface between the filler and the matrix, and system temperature. For example, Toulemonde et al. [13] observed the modulus distribution on a microscopic scale using AFM, clearly depicting the boundaries between white filler particles, dark gray matrix, and light gray interfaces. The evolution of Young’s modulus showed that the modulus in the interfacial region is about five times that of the matrix (5 MPa). Benedetto et al. [14] and Van et al. [15] studied the failure mechanisms of the binder Tepanol in HTPB/AP/Al systems under varying temperatures and tensile strain rates of the defect, showing significant effects of temperature on interfacial debonding. Lei et al. [16] used tensile tests on solid propellants to obtain parameters. They applied them in finite element models to predict the strain field and stress–strain response during interfacial debonding, validating that fracture initiation led to debonding. Hou et al. [17] conducted experimental and numerical simulation studies on debonding, nucleation, and crack propagation in HMX-MDB propellants, revealing three failure modes, with matrix fracture being the most prominent. Wubulaisan et al. [18] analyzed the damage mechanisms and mechanical responses under tensile and compressive loads, showing that interfacial characteristics play a more significant role in debonding than particle size in propellants containing multiple fillers.

Since adsorption processes and equilibrium states cannot be directly observed in experiments, MD simulations provide detailed structural and dynamic information at the atomic and molecular levels. MD simulations offer precise control and the ability to modify parameters, allowing for systematic studies of system behavior under extreme conditions such as high temperature and pressure, making them an effective tool for researching material structures and properties [19,20,21,22]. Defects in propellants, including filler, pore, crosslinking, and interfacial defects, are inevitable during production. MD simulations can assist in understanding the mechanical properties of defect-containing propellants [23,24,25,26,27,28].

The PBT/AP interface significantly influences the mechanical properties of propellants. However, most prior research has focused on the flawless AP crystalline surface, with fewer studies addressing the defective AP crystalline surface. This study employs MD simulations to investigate the interfacial adsorption behavior of PBT matrix–AP interfaces, focusing on the effects of interfacial defects and matrix crosslinking rates on propellant adhesion performance. The goal is to provide a theoretical contribution to understanding the PBT matrix–AP interaction mechanism and interfacial structural relationships. The paper consists of four sections. Section 2 details the PBT matrix–AP interfacial model, force fields, and simulation methods. Section 3 presents the analysis of interfacial strength, the adsorption behavior of the defective interfacial models, and the structural evolution results, followed by conclusions in Section 4.

## 2. Initial Models

**Figure 1:**
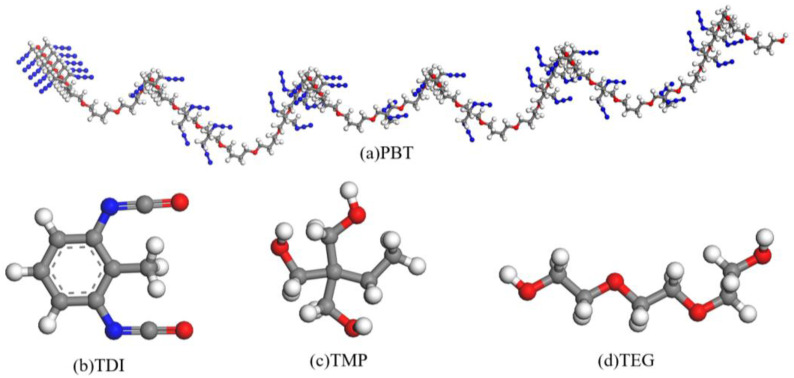
Molecular models of (**a**) PBT, (**b**) TDI, (**c**) TMP, and (**d**) TEG. Color scheme: grey (carbon), blue (nitrogen), red (oxygen), white (hydrogen).

The molecular models were constructed using the Materials Studio software, and MD simulations were performed within the Forcite and Amorphous Cell modules of Materials Studio. The crosslinking reactions were executed using Perl scripts. The PBT binder consisted of a 1:1 molar ratio copolymer of 33-bis(azidomethyl)oxetane and tetrahydrofuran, with TDI as the curing agent, TMP as the crosslinker, and triethylene glycol (TEG) as the chain extender. Figure 1 shows the ball-and-stick molecular models of PBT (a), TDI (b), TMP (c), and TEG (d).

**Figure 2:**
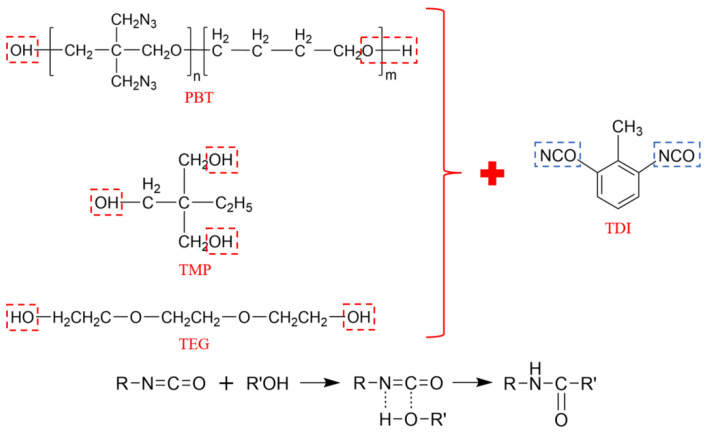
Crosslinking reaction mechanism.

The crosslinking reaction mechanism for the PBT-based azide composite propellant is shown in Figure 2 [2]. The hydroxyl groups on PBT, TMP, and TEG react with the isocyanate groups on TDI to form urethane linkages. TMP, having trifunctional groups, acts as a crosslinking agent, forming a branched network, while TEG serves as a chain extender with bifunctional groups.

To maximize the reaction between [-NCO] and [-OH], the molar ratio of [-NCO] to [-OH] was set to 1:1 in this study. Here, [-R] represents the hydrocarbon backbone of TDI, while [-R’] represents the hydrocarbon or alkyl chains of PBT, TMP, and TEG. The molar ratio of the components in the crosslinking reaction formula was set to PBT: TDI: TMP = 55:100:5:35. The molecular models were filled into a low-density periodic cubic box with an initial density of 0.3 g/cm^3^. The entire system was globally optimized before initiating the reactions. After eliminating high-energy regions, MD simulations were performed under an NPT ensemble using the COMPASSION force field, with a simulation time of 3 ns and a time step of 1 fs. The system reached its lowest energy structure at a final density of 1.15 g/cm^3^, which was used as the initial configuration before the crosslinking reaction.

**Figure 3:**
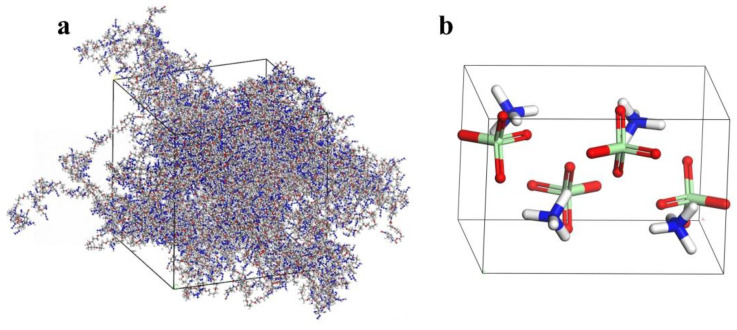
(**a**) Ball-and-stick model of the 90% crosslinked structure; (**b**) AP crystal unit cell stick model. Color scheme: cyan (carbon), blue (nitrogen), red (oxygen), white (hydrogen).

This study applied a probability-controlled bonding method to regulate the crosslinking rate by controlling the reaction radius. The C atom in [-NCO] and the O atom in [-OH] were set as reactive sites, and their reaction probability was set to 50% when their distance was below the target crosslinking radius. The formed bonds gradually increased as the crosslinking reaction progressed, raising the crosslinking degree. The crosslinking process was stopped once the target crosslinking degree was reached. Models at different crosslinking degrees (90%, 80%, 70%) were recorded throughout the reaction. A ball-and-stick model of the 90% crosslinked structure is shown in Figure 3a.

The AP crystal parameters used in this simulation were obtained from single-crystal neutron diffraction experiments [29]. These experiments showed that AP crystallizes in an orthorhombic unit cell with a space group of Pnma (62). The lattice parameters are a = 8.94 Å, b = 5.89 Å, c = 7.3 Å, and α = β = γ = 90°, with a density of 1.95 g/cm^3^. The (101) crystal plane, one of the most exposed surfaces of the AP crystal, was selected for model construction to elucidate the interfacial strength within the propellants. The AP crystal surface was cleaved along the (101) plane with a cutting depth of 13 Å, and a 13 × 7 × 13 supercell was constructed for periodic simulations.

**Figure 4:**
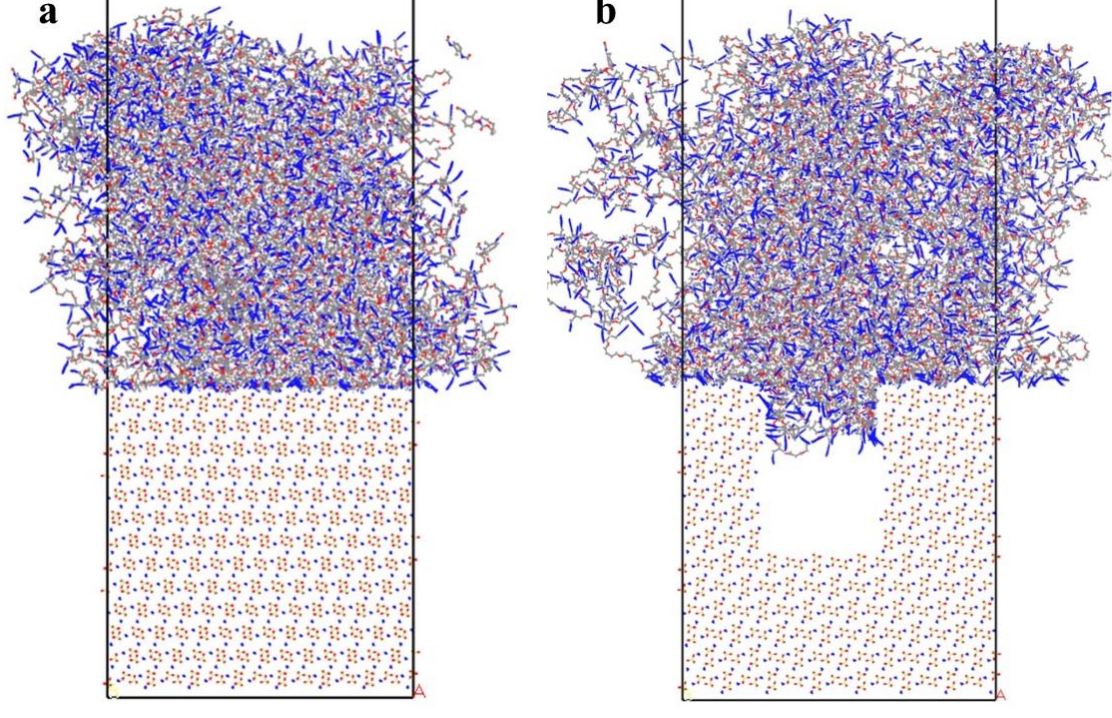
(**a**) Adsorption stick model of PBT matrix with crosslinking degrees of 80% and flawless AP interface; (**b**) adsorption stick model of PBT matrix with crosslinking degrees of 80% and defective AP. Color scheme: grey (carbon), blue (nitrogen), red (oxygen), white (hydrogen).

This study constructed two types of models: the first placed the PBT matrix, with different crosslinking degrees, on top of an intact AP surface to form a two-phase adsorption model (Figure 4a); the second placed the PBT matrix with an 80% crosslinking degree over an AP surface with rectangular defects to form a two-phase defective adsorption model (Figure 4b).

All simulations in this paper use the COMPASSII (Condensed-phase Optimized Molecular Potentials for Atomistic Simulation Studies II) force field. The COMPASSII force field is a model specifically designed for molecular dynamics simulations. It covers interactions from the atomic level to the molecular level. The COMPASSII force field, which combines quantum mechanics calculations with experimental data, accurately describes intermolecular interactions in various chemical environments. It has been successfully applied in simulations of explosives and propellants. The force field parameters of COMPASSII are obtained based on the fitting results of quantum chemistry, and also corrected based on experimental results [30,31,32,33,34,35,36,37].

## 3. Results and Discussion

### 3.1. Interfacial Adsorption Behavior

#### 3.1.1. Adsorption Behavior Without Defects

Periodic boundary conditions were applied in the x, y, and z directions, with a 60 Å vacuum layer added above the crosslinked layer to avoid interactions between the crosslinked layer and the periodic image of the AP layer [38,39]. MD simulations were then performed under periodic boundary conditions using the NVT ensemble at a temperature of 700 K, with a time step of 0.2 fs and a total simulation time of 8 ns. An Anderson thermostat was used to maintain temperature, and the Particle–Particle–Particle–Mesh (PPPM) method was employed for Coulomb interactions. Figure 4a shows the relaxed structure of the PBT matrix–AP interface after adsorption.

**Figure 5:**
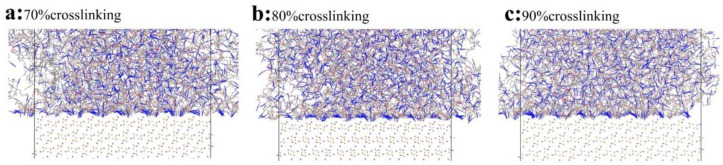
Enlarged adsorption modeling of PBT matrix and flawless AP interface with crosslinking degrees of (**a**) 70%, (**b**) 80%, and (**c**) 90%. Color scheme: grey (carbon), blue (nitrogen), red (oxygen), white (hydrogen).

In the adsorption structure (Figure 5), the PBT matrix is adsorbed on the AP surface, forming a tightly arranged lattice structure, indicating vdW interactions between AP and the PBT matrix molecules. Furthermore, the adsorption area near the interface shows a high concentration of nitrogen atoms (blue), with the azide chains of the PBT matrix primarily adsorbing onto the surface. Hydrogen bonds form between the nitrogen atoms of the azide chains in the PBT matrix and the atoms on the AP surface along the PBT-AP interface.

However, the PBT matrix at the far interface is more loosely distributed, with the azide chains evenly spread and no significant downward concentration, suggesting that interactions primarily occur near the interface. Two possible explanations are (1) that the interfacial adsorption strength between the PBT matrix and AP is relatively weak, and (2) the PBT azide crosslinker is a highly flexible molecular network with significant freedom of movement. Even after adsorption, chain segments may oscillate or sway freely, causing the local molecular chains to return to a looser arrangement.

#### 3.1.2. Adsorption Behavior with Defects

This section examines the effect of defects on interfacial adsorption behavior. We examine some of the critical issues of propellant interfacial defects by idealizing defects to represent possible samples in real interfaces. For comparison, defect models were constructed by removing portions of the AP layer to form defects with depths of 40 Å and widths of 20 Å, 30 Å, and 40 Å. As in previous models, a 60 Å vacuum layer was added above the crosslinked layer to prevent interactions with periodic images. MD simulations were performed under periodic boundary conditions with the NVT ensemble, a time step of 0.2 fs, and a simulation temperature of 700 K for 10 ns. The Anderson thermostat was used for temperature control, and the PPPM method was applied for Coulomb interactions. The adsorption structure is shown in Figure 4b.

**Figure 6:**
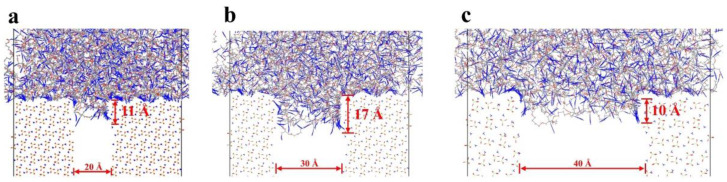
Enlarged adsorption modeling of PBT matrix with 80% crosslinking degree and AP with defect widths of (**a**) 20 Å, (**b**) 30 Å, and (**c**) 40 Å. Color scheme: grey (carbon), blue (nitrogen), red (oxygen), white (hydrogen).

The presence of defects has a significant effect on interfacial adsorption. Inside the defect surface, the azide chains (blue) are mainly adsorbed on the sides of the defect, increasing the overall interfacial contact area and enhancing interfacial adsorption capacity (Figure 6). The effective adsorption contact areas for three distinct defect widths were quantified using Perl scripting. The minimum interatomic separation between the PBT polymer atoms and the AP surface atoms was ascertained by initially identifying all atoms within the AP surface boundary, employing a cutoff radius of 4 angstroms (Å). Specifically, interatomic distances below this threshold were designated as contact points. Subsequently, the statistical contact area was derived from the spatial extent of the AP surface atoms that were in contact. The data revealed that the effective adsorption contact areas corresponded to 311.4, 484.6, and 207.1 square angstroms (Å^2^) for defect widths of 20 Å, 30 Å, and 40 Å, respectively. Comparing the adsorption models with defect widths of 20 Å, 30 Å, and 40 Å, it is evident that the adsorption depth is most significant for the 30 Å defect, while the depths for the 20 Å and 40 Å defects are smaller. This phenomenon may be due to two factors: (1) although interaction forces exist within the inner walls of the defect, the minor 20 Å defect does not provide enough space for the larger PBT crosslinking matrix to form sufficient adsorption contacts, and (2) while the 40 Å defect provides enough space to accommodate the PBT matrix, the adsorption ability of the intact surface is weakened, resulting in less adsorption onto the defect’s inner walls. These results indicate that defects lead to the matrix’s adsorption onto the defect’s inner walls, which increases the adsorption surface area. If depth is not considered (adsorption does not reach the bottom of the defect), a defect of appropriate size increases the adsorption surface area, thus enhancing interfacial adsorption. However, substantial defects reduce adsorption strength, as the adsorption surface on the inner walls of the defect cannot compensate for the loss of adsorption on the flat surface.

**Figure 7:**
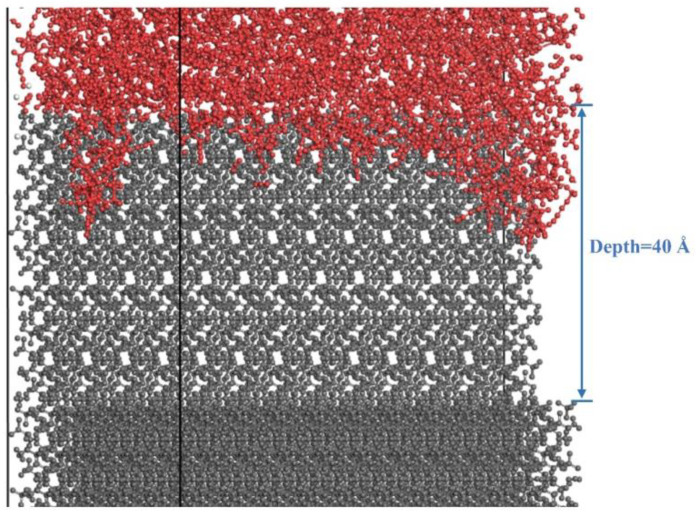
Magnified side-perspective cross-section views of the PBT matrix with 80% crosslinking degree adsorbed onto AP defects with varying widths (30 Å). Color scheme: red (PBT matrix), grey (AP surface).

Figure 7 presents a magnified side view of the interfacial adsorption of an 80% crosslinking rate PBT matrix with an AP defect. The adsorption inside the defect is sparse, with weak adsorption inside and at the surface, showing only a tiny number of azide chains tightly packed within the defect. The amount of azide chain adsorbed in the body showed non-uniform distribution characteristics, mainly occurring at the two ends of the defect cross-section. This was manifested as higher adsorption at the two ends near the filler and lower adsorption in the center region of the defect. This indicates that defects lead to reduced adsorption in the center, weakening overall interfacial adsorption strength.

### 3.2. Interfacial Binding Energy

Interfacial binding energy directly reflects the strength of interactions at the PBT matrix–AP interface. The greater the absolute value of the binding energy, the stronger the interaction and the more stable the interface. To investigate the effect of crosslinking degree on interfacial adsorption, the binding energy was calculated as follows [40,41]:

(1) $$E_{bind}=(E_{Mat}+E_{AP})-E_{Total}$$

where $E_{Total}$ is the system’s total energy, $E_{Mat}$ is the energy of the PBT matrix, and $E_{AP}$ is the energy of the AP crystal.

**Figure 8:**
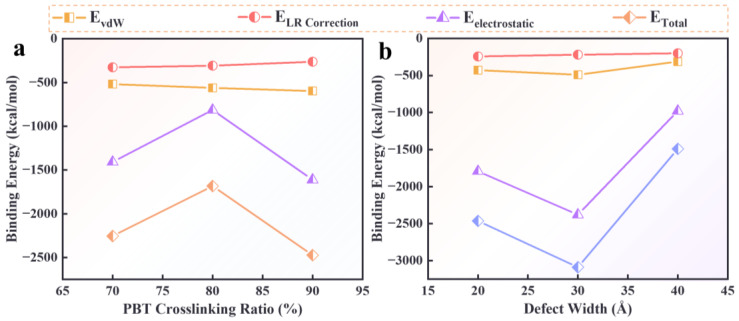
(**a**) Relationship between interfacial binding energy and crosslinking degree at the PBT-AP flawless interface. (**b**) Relationship between interfacial binding energy and defect width at the PBT-AP interface with an 80% crosslinked PBT matrix.

The interfacial binding energies of the PBT matrices with 70%, 80%, and 90% crosslinking degrees were calculated, as shown in Figure 8a. The results indicate that the interfacial binding energies are −2254.23 kcal/mol, −1683.201 kcal/mol, and −2472.62 kcal/mol for crosslinking degrees of 70%, 80%, and 90%, respectively.

The order of interfacial adsorption strength is $E_{90\%}$ > $E_{70\%}$ > $E_{80\%}$. The lower binding energy at 80% crosslinking is due to incomplete crosslinking reactions, resulting in fewer functional groups (such as [-NCO] and [-OH]) capable of interacting with the AP surface. At 90% crosslinking, the formation of a crosslinked network enhances the adsorption capability, resulting in stronger interactions than when only individual functional groups were present. Therefore, incomplete crosslinking reactions weaken the atomic interaction capacity, which is macroscopically expressed as weakening the interfacial adsorption capacity, making debonding more likely to occur.

Figure 8b shows the binding energy of the PBT matrix (80% crosslinked) on AP surfaces with defects of varying widths (20 Å, 30 Å, and 40 Å). The binding energies for defect widths of 20 Å, 30 Å, and 40 Å are −2463.93 kcal/mol, −3088.90 kcal/mol, and −1490.60 kcal/mol, respectively. The order of interfacial adsorption strength is $E_{30\;Å}$ > $E_{20\;Å}$ > $E_{\mathrm{Flawless}}$ > $E_{40\;Å}$, where $E_{\mathrm{Flawless}}$ represents the binding energy at the interface between the PBT matrix and the non-defective AP at a crosslinking ratio of 80%. This result indicates that substantial defects weaken interfacial adsorption. However, for the 20 Å and 30 Å defects, the increased adsorption surface area compensates for the loss in flat surface area, resulting in stronger adsorption than the flawless interface. For the 40 Å defect, the inner wall adsorption is insufficient to compensate for the reduced surface area, leading to a drop in binding energy. These results suggest that defects with widths between 30 Å and 40 Å provide the most substantial adsorption, consistent with the adsorption behavior discussed in Section 3.1.2.

Non-bonded interaction energies were analyzed to further investigate the underlying mechanisms of binding energy changes under different adsorption conditions. The components of the interaction energy include vdW interaction energy (*E*_vdW_), electrostatic interaction energy (*E*_electrostatic_), and long-range correction energy (*E*_LRCorrection_). The results show that the changes in interfacial binding energy are primarily influenced by electrostatic interactions (*E*_electrostatic_), which vary with defect width. In contrast, vdW interactions and long-range corrections fluctuate slightly and exhibit no significant trends.

### 3.3. Radial Distribution Function (RDF)

The interfacial structure was characterized using the RDF to further elucidate the interactions between different components on the AP surface. The RDF provides insights into the microscopic structure of materials, representing the probability of finding a particle at a given distance from a reference particle, normalized to the probability under uniform distribution assumptions. The RDF is defined as follows:

(2) $$g_{AB}(r)=\frac{1}{\rho_{AB}4\pi r^{2}\delta_{r}}\frac{\sum_{t=1}^{K}\sum_{j=1}^{N_{AB}}\Delta N_{AB}(r\to r+\delta_{r})}{N_{AB}\times K}$$

where $g_{AB}(r)$ is the ratio of the probability density of B(A) atoms occurring at a distance around an A(B) atom to the probability density of a random distribution (A and B can be the same atom), $\Delta N_{AB}$ is the number of B (or A) atoms in the range $r\to r+\delta_{r}$, K is the total time step, $\delta_{r}$ is the distance interval, and $\rho_{AB}$ is the average number density.

**Figure 9:**
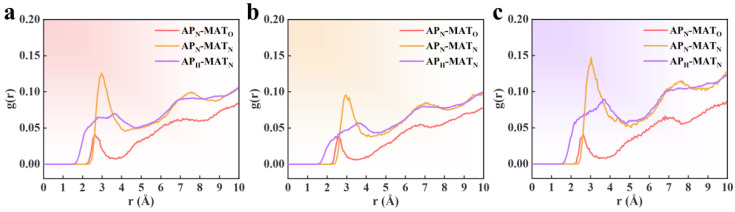
RDF of atom pairs at the PBT-AP flawless interface with crosslinking degrees of (**a**) 70%, (**b**) 80%, and (**c**) 90%.

**Figure 10:**
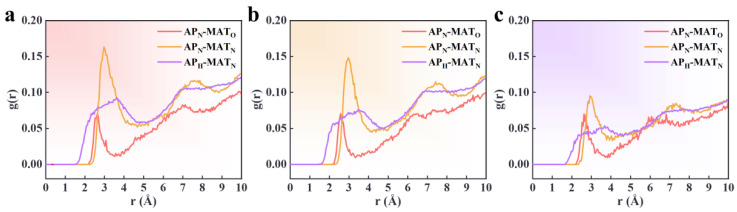
RDF of atom pairs at the PBT matrix–AP interface with defect widths of (**a**) 20 Å, (**b**) 30 Å, and (**c**) 40 Å.

Intermolecular RDF can analyze interactions, including hydrogen bonding and vdW forces. Hydrogen bond interactions occur in the range r = 1.1–3.3 Å, while solid vdW forces operate within r = 3.1–5.0 Å, and weaker vdW forces occur at distances greater than 5.0 Å [42,43]. In this study, the N and H atoms in AP are denoted as AP_N_ and AP_H_, and the O and N atoms in the PBT matrix are denoted as MAT_O_ and MAT_N_, respectively. Figure 9 and Figure 10 show the RDF curves for different atom pairs under varying conditions. The x-axis represents the distance between atom pairs in Å, and the y-axis represents the probability density.

The RDFs for the PBT matrix adsorbed onto a flawless AP surface at 70%, 80%, and 90% crosslinking degrees show similar trends, with the AP_N_-MAT_N_ interaction being dominant and the AP_N_-MAT_O_ interaction being the weakest. The first peak for the AP_N_-MAT_O_ RDF occurs at approximately 2.6 Å, indicating the presence of hydrogen bonds. However, the relatively low peak height suggests the interaction is not particularly strong. The first RDF peak for AP_N_-MAT_N_ occurs at around 3.0 Å, indicating hydrogen bonding interactions. The AP_H_-MAT_N_ RDF exhibits a weak peak at around 3.7 Å, suggesting robust vdW interactions between these atoms. Additionally, the RDF curves show peak fluctuations at 7–7.5 Å, but the fluctuations are insignificant, suggesting some interactions at longer distances. This is due to the poor adsorption capacity of AP to the PBT matrix, and there is also a distal part of the interface that is not fully adsorbed.

For the PBT matrix adsorbed onto a flawless AP surface, the RDF peak heights of AP_N_-MAT_N_ are 0.125, 0.0959, and 0.147 for 70%, 80%, and 90% crosslinking degrees, respectively. The RDF peak heights for AP_H_-MAT_N_ are 0.07, 0.055, and 0.089, respectively. The number of interfacial interaction atom pairs is the lowest at 80%, the highest at 90%, and the next highest at 70%. The results show that an incomplete crosslinking reaction leads to a decrease in the interfacial adsorption capacity, manifested by the fact that debonding between AP and the PBT matrix is more likely to occur. This is consistent with the binding energy calculations, which are smallest at the 80% crosslinking rate, largest at the 90% crosslinking rate, and the next highest at the 70% crosslinking rate, demonstrating that the target atom pairs of vdW interactions express adsorption affinity at the interface.

In the RDF analysis of the PBT matrix adsorbed onto the defective AP surface, evident variations are observed in the AP_N_-MAT_N_ and AP_H_-MAT_N_ atom pairs. Figure 10 shows the RDF curves for defect widths of 20 Å, 30 Å, and 40 Å. The RDF peak heights for AP_N_-MAT_N_ are 0.147, 0.162, and 0.0952, respectively, while those for AP_H_-MAT_N_ are 0.074, 0.091, and 0.053. These results indicate that as the defect width increases, the number of interacting atom pairs between AP_N_ and MAT_N_ initially increases and then decreases. The AP_H_-MAT_N_ RDF shows a gradual disappearance of the hydrogen bond interaction peak as the defect width increases, indicating a weakening of the adsorption strength at larger defect widths. This suggests that defect width is crucial in determining interfacial adsorption strength, with adsorption capacity dropping significantly at defect widths of 40 Å.

Compared to the RDF of the PBT matrix on a flawless AP surface (80% crosslinked), where the peak height for the AP_N_-MAT_N_ RDF is 0.0959, the defect widths of 20 Å and 30 Å show an increase in RDF values. As analyzed in Section 3.1.2, this can be attributed to the increased adsorption surface area inside the defect, compensating for the lost flat surface area and enhancing interfacial adsorption strength.

Additionally, the RDF values in Figure 9 and Figure 10 are relatively low, indicating that the density of interacting atoms is relatively low within the selected distance range. Three factors can explain this:

1. Non-uniform adsorption: Even in cases of complete interfacial adsorption, adsorption may be localized and non-uniform, concentrated in specific regions. If adsorption only occurs in localized areas, the interaction between reference and target atoms may be limited to small zones, resulting in low RDF values over more considerable distances. RDF reflects an average over the entire simulation area rather than being restricted to localized adsorption sites.
2. Weak vdW and electrostatic interactions: The dominant interactions between the PBT matrix and AP are relatively weak vdW and electrostatic forces. The lack of atomic solid correlations leads to fewer neighboring atoms within the specified range, resulting in a lower RDF. This weak interaction manifests as a lack of spatial correlation the between atoms in the MD simulations, causing the RDF curves to remain low at short distances.
3. Flexibility of the azide chains: The flexible azide chains in the PBT crosslinked network may result in loose chain interactions, with large fluctuations in the relative positions of atoms. This flexibility weakens interatomic interactions, leading to low RDF values. The random arrangement of flexible chains means that the RDF curves do not exhibit pronounced peaks even when adsorption occurs.

### 3.4. Temperature Effects

**Figure 11:**
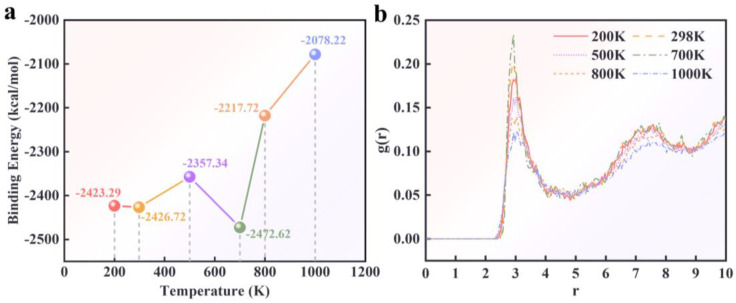
(**a**) Effect of temperature on the binding energy of the PBT matrix–AP interface. (**b**) Effect of temperature on radial distribution function (RDF) of N-N atom pairs.

Temperature is a critical factor influencing the performance of rocket propellant fuels, as extreme temperatures can lead to mechanical failure or even structural decomposition. The effect of temperature on interfacial adsorption strength was investigated to explore the temperature sensitivity of the PBT azide matrix–AP interface. Binding energies were calculated for six different temperatures—200 K, 298 K, 500 K, 700 K, 800 K, and 1000 K—using the PBT matrix–AP interface model with 90% crosslinking. Each simulation was repeated at each temperature, and the average binding energy was recorded. Figure 11a shows the temperature-dependent binding energy curve for the PBT matrix–AP (101) interface model with 90% crosslinking.

The results show that the interfacial binding energy fluctuates with increasing temperature. It remains relatively stable at low and ambient temperatures, with minor fluctuations. However, at 700 K, the binding energy reaches its maximum, indicating the most substantial interfacial adsorption. As the temperature increases beyond 700 K, the binding energy decreases sharply, reflecting a significant reduction in adsorption strength at 800 K and 1000 K. This suggests that the PBT matrix–AP interface becomes increasingly unstable at high temperatures, potentially leading to debonding or mechanical failure.

To elucidate the temperature-induced interfacial structural dynamics at defect interfaces, RDF analysis was conducted in this study. The RDFs of the 30 Å wide defect interfaces were computed across a range of temperatures: 200 K, 298 K, 500 K, 700 K, 800 K, and 1000 K, as depicted in Figure 11b. The RDF, detailed in Section 3.3, indicates that the AP_N_-MAT_N_ atom pair constitutes the predominant variable influencing the interfacial structure. Consequently, the temperature-dependent analysis focuses on the RDF fluctuations associated with the AP_N_-MAT_N_ atom pair. The findings reveal that the peak RDF values of the AP_N_-MAT_N_ atom pair at 700 K are maximized. Beyond 700 K, the impact of the AP_N_-MAT_N_ atom pair diminishes with increasing temperature. The peak RDF values associated with the AP_N_-MAT_N_ atom pairs exhibit a temperature-dependent variation, mirroring the observed trend in the interfacial binding energy alterations, as previously discussed.

## 4. Conclusions

In this study, all-atom molecular dynamics simulations were employed to investigate the adsorption behavior and mechanisms of PBT matrix–AP systems, both with and without defects. The effects of different PBT matrix crosslinking degrees, AP defects, defect sizes, and temperature on interfacial adsorption, binding energy, and RDF were analyzed. The conclusions drawn from the findings are summarized below:

1. The interfacial adsorption strength between the PBT matrix and AP surface is relatively weak. The adsorption state of the PBT matrix is loose for all three crosslinking rates. Defects on the AP surface result in the adsorption of the PBT matrix onto the defect walls, with maximum adsorption observed at intermediate defect sizes.
2. The interfacial binding energy calculations for flawless AP surfaces with varying crosslinking degrees show the following trend: *E*_90%_ > *E*_70%_ > *E*_80%_. For AP surfaces with different defect sizes at 80% crosslinking, the binding energy shows the following trend: $E_{30\;Å}$ > $E_{20\;Å}$ > $E_{40\;Å}$. These results indicate that incomplete crosslinking reduces adsorption strength, while appropriately sized defects enhance interfacial adsorption.
3. The RDF analysis reveals the mechanism behind adsorption strength for flawless and defective interfaces, which aligns with the binding energy results. The AP_N_-MAT_N_ atomic interaction is the primary factor influencing the adsorption strength at the PBT-AP interface. Additionally, the RDF values indicate non-uniform or localized adsorption with relatively weak interatomic binding.

## Data Availability

Data will be made available on request.

## Abbreviations

MD	molecular dynamics
AP	ammonium perchlorate
PBT	poly(3,3-bis-azidomethyl oxetane)-tetrahydrofuran
RDF	radial distribution functions
TDI	toluene diisocyanate
TMP	trimethylolpropane
TEG	triethylene glycol
PPPM	Particle–Particle–Particle–Mesh
vdW	van der Waals
